# Personal goal-setting among women living with breast cancer: protocol for a scoping review

**DOI:** 10.1186/s13643-018-0794-6

**Published:** 2018-08-28

**Authors:** Andrea Chow, Justin Presseau, Iris Perelman, Lindsey Sikora, Dean Fergusson

**Affiliations:** 1 School of Epidemiology and Public Health, University of Ottawa, Ottawa, Canada; 2 Clinical Epidemiology Program, Ottawa Hospital Research Institute, Ottawa Canada and School of Epidemiology, Public Health and Preventive Medicine, University of Ottawa, Ottawa, Canada; 3 Ottawa Hospital Research Institute, Ottawa, Canada; 4 Health Sciences Library, University of Ottawa, Ottawa, Canada

**Keywords:** Goal-setting, Personal goals, Breast cancer, Scoping review

## Abstract

**Background:**

Breast cancer and its treatment can have many physical and psychological effects on affected women. Women’s personal goals may provide insight into their priorities and motivations in the context of breast cancer. Incorporating personal goal-setting into support and care interventions may have an effect on psychological well-being. This protocol describes our scoping review methods, the aim of which is to examine and map the existing evidence on personal goal-setting among women with a breast cancer diagnosis.

**Methods:**

Our scoping review will search for published, full-length articles, where personal goal-setting is a major component of the study, and the study population is females with breast cancer. MEDLINE, PsycInfo, CINAHL, EMBASE, the Cochrane Library, and AMED databases will be searched. Two independent reviewers will conduct all screening and extract data. Descriptive information about the studies, participants, any interventions, measurement tools, outcomes, and results will be reported.

**Discussion:**

The results from this review will chart the literature, contributing to optimizing the incorporation of personal goal-setting approaches into effective interventions for the care and support of women with breast cancer.

**Electronic supplementary material:**

The online version of this article (10.1186/s13643-018-0794-6) contains supplementary material, which is available to authorized users.

## Background

*Personal goals* are an individual’s cognitive expressions of desired states or processes which provide directional motivation towards a state [1, 2], i.e., they are something a person wants to aim for, maintain, or avoid. Everyday life is characterized by the pursuit of personal goals, such as normative goals (for example, “keep meeting my sister for lunch every Sunday”) and self-defining idiosyncratic pursuits (for example, “read one biography every week”), combining to describe important expressions of life motivations [3]. *Personal goal setting* is the action of selecting or establishing personal goals and provides motivation for further action towards pursuing goals [1, 4, 5]. The personal goals we set and pursue are influenced by individual and contextual factors, both stable and dynamic [1, 6]. For example, individual traits such as extraversion and conscientiousness are predictors of the types of goals set by young adults [7]. The diagnosis of any cancer or serious illness can change the goals that people pursue by prompting the addition of new goals and the disengagement from existing goals, or by shifting a person’s focus from extrinsic to more intrinsic (for example, health-related) goals [8–10]. Cancer and its treatment can also limit an individual’s time and physical and mental energy to pursue personally important goals [8, 11]. For instance, for women with breast cancer, treatment can have many side effects including fatigue, pain and physical limitations, premature menopause, and negative effects on cognitive functioning [12–14]. Women may also experience anxiety, distress, or mental health concerns as a result of the diagnosis and throughout the treatment. Indeed, a higher burden of breast cancer-related physical symptoms has been associated with a reduced ability to pursue goals and with psychological distress [15]. While generally the disruption of goal pursuit can reduce well-being and the ability to engage in new goals is positively associated with subjective well-being and better psychological outcomes [16], the dynamics of such goal pursuit with a major life event such as breast cancer diagnosis and treatment is less clear. Supporting women with breast cancer to set and pursue goals that are meaningful may result in outcomes that contribute to a higher quality of life. Stakeholders tasked with design and delivery of interventions and programs of care that can support women with their personal goal pursuit would benefit from greater clarity in how personal goal setting has been approached in this population. While a previous systematic review collated research on the life goals of people living with any type of cancer [9], to our knowledge, there is no assessment of the literature specifically focussed on goal-setting among women with breast cancer.

Women diagnosed with breast cancer face many challenges similar to people with a different cancer diagnosis, but they are also a unique population in several ways important to personal goal-setting and those developing support for them. Breast cancer overwhelmingly affects women. Gender, which is shaped by individual, social, and cultural contexts, may predict some goals: women may tend to value goals for social harmony more than men, who tend to value economic achievement goals more than women [7]. Among younger women, cancer diagnoses are most likely to be of the breast than any other cancer [17, 18]. In healthy populations, the content of personal goals tends to change with age, and perceived control over health goals decreases with age [19, 20]. Because breast cancer affects more younger women than other cancers, illness and age may potentially influence differences in personal goal selection and factors associated with their pursuit. Furthermore, this is an active area of research and we have informally identified several additional studies on the goals of women with breast cancer published since Hullmann et al. conducted the search for their study in 2014 [21–27].

In this protocol, we present our methods for conducting a scoping review. The objective of our scoping review is to examine and map the existing literature on personal goal-setting among women with a breast cancer diagnosis, with the aim to address four research questions:

1. How many studies have examined personal goal-setting among women with breast cancer and what were the characteristics of these studies?
2. In studies that involved goal-setting as part of an intervention, what were the characteristics of the interventions used in these studies; what comprised the intervention; who delivered the intervention; and when, where, and how was the intervention delivered?
3. What instruments were used in these interventions to elicit and evaluate the personal goals of women with breast cancer?
4. What were the primary results of the studies that have used validated instruments?

## Methods

This protocol was developed following guidelines for planning and conducting systematic and scoping reviews recommended by the Joanna Briggs Institute and the Cochrane Collaboration [28–31]. A completed PRISMA-P checklist is presented in Additional file 1. This protocol is not registered on PROSPERO.

### Search strategy

To minimize missed studies and bias, several electronic databases will be searched, from inception to the present day: MEDLINE (OVID interface), PsycINFO (OVID interface), EMBASE (OVID interface), CINAHL (Ebsco interface), AMED (OVID interface), and the Cochrane Library (Ovid interface, composed of seven databases: Cochrane Database of Systematic Reviews; ACP Journal Club; Database of Abstracts of Reviews of Effects, current issue; Cochrane Central Register of Controlled Trials, current issue; Cochrane Methodology Register, current issue; Health Technology Assessment, current issue; and NHS Economic Evaluation Database, current issue).

The trials register, clinicaltrials.gov, will be searched for additional trials. To ensure literature saturation, the reference lists of included studies and relevant reviews identified through the search will be scanned for further citations. Any relevant conference abstracts identified in the search will be highlighted for keywords and electronic databases and Google Scholar will be searched to identify any full-length articles related to the abstract. Other gray literature will not be searched.

To identify published literature, this review will employ a strategy guided by recommendations from the Joanna Briggs Institute Reviewers Manual and the Cochrane Collaboration [28, 30, 31]. Literature search strategies were developed using medical subject headings (MeSH) and text key words related to breast cancer, goals, and goal-setting adapted from a systematic review on goal-setting in rehabilitation [32]. The outcomes will not be restricted in the search strategy. A literature search strategy for MEDLINE was developed with the aid of a health sciences librarian with experience in scoping review searching, Lindsey Sikora (LS). A draft of the MEDLINE strategy is outlined in Additional file 2. This strategy, once finalized, will be adapted to the syntax and subject headings of the other databases by AC and checked by LS. After articles are screened for inclusion in two stages, the reference list of all included papers will be searched for additional studies.

No study design, publication status, date, or language limits will be imposed on the search, although only studies in English will be eligible for inclusion, due to resource limits. Although conference abstracts, theses, and dissertations will not be included in our review as outlined in the eligibility criteria below, they will be searched for to ensure literature saturation as described above. Databases will be searched for articles published at any time up until June 2017 (up until and including the date of the initial search).

### Data management

Literature search results will be uploaded to and merged using Endnote X7 software. Inclusion and exclusion of citation abstracts in stage 1 screening and articles at stage 2 screening will be tracked using *Covidence*.

### Selection process

Two reviewers will conduct a two-stage screening process to minimize bias and errors and increase rigor. After the literature search is conducted and duplicates removed, two reviewers will independently and in duplicate screen the titles and abstracts identified in the search strategy detailed above. Each title will be screened using a screening guide. Titles will be retained if they appear to meet the inclusion criteria of if it is uncertain if they do.

Full-text articles will be obtained for all titles retained from stage 1 screening. If a full-text article (excluding conference abstracts) from an electronic database cannot be located online, the study authors will be contacted to request a copy of the article. A maximum of two email attempts over 2 weeks will be made. If an article cannot be obtained from the authors, or must be purchased to be obtained, it will be excluded and the reason documented. If the text of a conference abstract cannot be obtained online, a related full-length article will not be searched for. For all grey literature, including theses or dissertations, if the full-text article cannot be located online, the citation will be excluded and the reason documented. At stage 2 screening, two reviewers will independently and in duplicate screen all full-text articles, examining them for compliance with the eligibility criteria of this review. Disagreements will be resolved through discussion. If agreement cannot be made, a third party (DF or JP) will arbitrate.

To avoid inclusion of duplicate publications of the same study, the list of included studies at stage 2 screening will be examined for: the same author names, locations or settings, interventions, number of participants, study dates, and study duration. If duplicate publications are identified, they will be removed from the list of included studies.

### Eligibility criteria

Inclusion and exclusion criteria are summarized in Table 1.

**Table 1:** Eligibility criteria

	Inclusion	Exclusion
Study design	Primary studies of any design	Systematic reviews
Population	Females with a diagnosis of breast cancer	Males, studies where multiple cancers are studied and outcome data for women with breast cancer cannot be extracted
Intervention/exposure	Examines personal goal-setting as major component of the intervention; involves the participant in setting goals	Personal goal-setting is not a major intervention component; examines organizational/group goals, participant preferences or life values; does not involve the participant in setting goals
Outcomes	All outcomes	n/a
Language	English	Anything other than English
Publication status	Published, full-length articles	Commentaries, letters, books, review articles, conference abstracts, theses or dissertations
Other	All study dates, length of follow-up, setting	n/a

#### Types of study design

Primary studies of any design (e.g., experimental, observational, qualitative, and cross-sectional) will be included. A broad range of studies is desired to meet this review’s scoping objective. Studies where the primary purpose was to develop or validate an outcome measurement tool will be included, as this study is interested in identifying instruments used to set and evaluate participants’ personal goals. Systematic reviews will be excluded.

#### Types of participants

This review will include studies involving participants who had ever received a diagnosis of breast cancer, regardless of time since diagnosis. Survivorship is considered a phase in the cancer experience, and cancer recurrence and mortality remain a possibility and fear. Only the female sex will be included; males will be excluded. Participants may be of any age. Any breast cancer stage or severity will be included. Women at any stage (planning, undergoing, completed) of any cancer treatment (chemotherapy, radiation, surgery, alternative therapies) will be included. Women who have decided not to pursue cancer treatment (for example, because of personal beliefs, or because their care is palliative) will be included. They can still access cancer care and support, and therefore, understanding participant involvement in goal-setting is relevant to this population. Studies that include other cancer sites (for example, prostate, lung) will be included if data on the outcomes for women with breast cancer can be extracted. Otherwise, studies where breast cancer is one of multiple cancer sites studied will be excluded.

#### Types of interventions

For studies identified that include the description of an intervention being evaluated, such interventions of all included studies must include either the identification of participants’ existing personal goals or the setting of new goals for the participant as a major component of the intervention. To be a major component, the article’s introduction should present a justification or theoretical basis for how goal-setting is expected to affect the outcome. Studies where goal-setting is not a major component of the intervention will be excluded. Studies examining personal goal-setting in any life domain (e.g., relationships, occupational, or health) will be included. Studies which examine organizational or group goal interventions, participant preferences (where participants select or rank goals from a provided list), or life values will be excluded. In studies involving the evaluation of interventions where the intervention involves setting new personal goals, studies that involve participants in goal-setting, either by having participants set the goals or having them set the goals jointly with someone else (e.g., a health worker or practitioner, a family member, researcher) will be included. Studies in which someone other than the participant (e.g., study researchers, health professionals) provides parameters for goals (e.g., goals for treatment, goals over the next month) will be included, as long as the participant is involved in setting the actual goals. Although goals for treatment may be elicited in order to inform treatment plans, we include them because they may become a part of an individual’s personal goal system [33] due to the nature of a cancer diagnosis and journey. Studies where goals are set by someone other than the participant without their involvement, or where someone other than the participant sets or recommends an overall goal, followed by participant involvement in setting smaller, intermediate goals towards the overall goal, will be excluded. In studies where the intervention/exposure involves setting new personal goals, the personal goals set must be articulated, either verbally or written during the intervention. Studies that only encourage or provide training or support for personal goal-setting, without setting personal goals in the study, will be excluded.

#### Types of outcomes

All outcomes reported will be included in the review. Measures can be subjective (e.g., sleep loss reported by the participant) or objective (e.g., sleep loss measured by sleep monitor). This review will distinguish primary outcomes of a reported study from other outcomes.

#### Language

Only studies published in English will be included, due to the linguistic abilities of the reviewers. Studies in any other language will be excluded.

#### Publication status

Only published, full-length articles will be included. Commentaries, letters, books, review articles, theses, dissertations, and conference abstracts will be excluded.

#### Other

There will be no restrictions on date of study or length of follow-up time. All clinical and non-clinical settings (for example, home care, community institutions) will be included. Care of women with cancer does not take place exclusively in a clinical setting, and comprehensive evidence of interventions is desired.

### Data extraction

This review will record key information from included articles in a Microsoft Excel data extraction form designed a priori. Two reviewers will independently extract data to minimize errors. The data abstraction form will be piloted to reduce potential errors in collecting data and determine if the form extracts the desired data. AC will review the objectives, methods, results, and discussion section of each article to identify any important information not extracted. The form will then be refined, re-piloted, and finalized, and then full data extraction conducted. Any modifications to the form will be documented in the final report.

If an article is missing information about the intervention, outcomes, or results, this will be noted in the data extraction. No data will be imputed. If duplicate publications are identified during data collection, they will be removed. If there are multiple reports from a single study, data from the study will be collated either by extracting data from each study separately and then combining the information from multiple data abstraction forms, or extracting data from all articles into one single data abstraction form, depending on the form of each article.

### Data items

Planned variables to be extracted in this review are outlined in Table 2 (subject to change, key information will be identified in an iterative process). Data extracted will include participant information, study information, methodology, intervention/exposure details, outcome measures, and all reported results.

**Table 2:** Planned variables to be extracted in the scoping review

General study details	Study ID number, lead author, title, journal, year of publication, type of publication, information source, primary and secondary purposes
Study characteristics	Study design, study duration, pilot/feasibility study (y/n), number of study arms, covariates (definition and measurement methods)
Participants	• Total number, setting, inclusion and exclusion criteria
	• Participant characteristics at baseline: for each study, average age (years, mean and standard deviation [SD]), sex (%), country, cancer treatment received, breast cancer treatment stage, time since diagnosis (or time since treatment, if treatment was completed)
	• *If available:* breast cancer stage, race or ethnicity, socioeconomic status
Interventions/exposures and comparators	• Total number of intervention/exposure and comparison groups, number of participants in each group
	• For each intervention/exposure and comparison group: justification for goal-setting, type of goal setting used (BCT), materials and procedures used, who administered the intervention/exposure/comparison, training to deliver goal-setting, mode of delivery, location, timing of delivery, duration of intervention/exposure, any tailoring of the intervention, any modifications, techniques to support goal pursuit, co-interventions (if any), intervention adherence or fidelity—who and how assessed, and results of assessment
	• For each intervention/exposure: participant involvement in personal goal-setting, new or existing goals identified, any parameters given for goal-setting.
	• For each comparison (if applicable): whether personal goal-setting was part of the intervention, participant involvement (if any) if goals were set
Instruments used in goal-setting	Type of instrument used, construct validity (if reported), concurrent validity (if reported), validated with women with breast cancer, feasibility (if reported)
Outcomes	• List of outcomes and time points (a) collected, (b) reported. Identification of study’s primary outcome
	• Data collection method: quantitative or qualitative
	• For each outcome: outcome definition (narrative plus name of scale or diagnostic method)
	• For scales: validity, upper and lower limits, direction of benefit
Results	For each quantitative outcome: sample size, number of missing participants, reasons for loss to follow up, summary data for each group (2 × 2 table for dichotomous data, means and SDs for continuous data), estimate of effect for difference between groups (or change in baseline and final scores for single-arm studies), confidence intervals, *p* value

The TIDieR checklist will be used to guide extraction of data about interventions [34]. We will extract information on goal-setting processes in study interventions, including their justification. For interventions which set new personal goals, intervention descriptions will be extracted as described in the papers, then characterized by coding using the Goals and Planning category (cluster 1) of the Behavior Change Technique Taxonomy [35], a classification system of behavior change techniques. We will also extract information on intervention techniques to support participants to successfully pursue their goals, and the validity of instruments used to identify and rate personal goals. We expect that, where validation has occurred, we will find evidence of construct validity and potentially concurrent validity. Given the individualized nature of goal-setting, we do not expect to find evidence of criterion validity. We plan to extract information on the populations with which identified instruments have been validated.

### Data synthesis

The number of studies identified and selected at each stage of the scoping review, along with the number of articles excluded at level 1 and 2 screening, and the reasons for exclusion at level 2 screening, will be presented in a PRISMA flow diagram [32]. Results will be summarized in table form and discussed in more depth in narrative form to address each of the four research questions. Results will be grouped conceptually, by study characteristics, participant characteristics, study objectives, intervention/exposure (if applicable), instruments used to identify personal goals, outcomes measured and time points, and results. This review will present summaries of these categories, with a particular focus on studies’ interventions or exposures, goal-setting techniques, including whether studies defined parameters for goals, and reported results, including quantitative measurements of associations (mean differences for scores by validated questionnaires, risk ratios or odds ratios for dichotomous outcomes) if applicable. Additional groups may be identified during the extraction of results.

## Discussion

By charting the literature on personal goal-setting among women with breast cancer, the results from this scoping review will contribute aggregated knowledge on the personal goals of women with breast cancer that can help to identify personal motivations that are important to individual women. This knowledge can be helpful for the development of new interventions or the optimization of existing ones, as part of appropriate treatment and care plans for women with breast cancer and survivors. Such care could help women to shift or adapt new goals to facilitate the likelihood of successful goal pursuit, improve psychological outcomes, and improve experiences of cancer care and treatment.

## Additional files

Additional file 1: PRISMA-P checklist. Completed checklist of recommended items to include in a systematic review protocol. (DOCX 33 kb)

Additional file 2: Medline search strategy. The search strategy to be used in the MEDLINE database. (DOCX 73 kb)

## Abbreviations

BCT: Behavior Change Taxonomy
PRISMA: Preferred Reporting Items for Systematic Reviews and Meta-Analyses
TIDieR: Template for Intervention Description and Replication
