# Supplementary material for: Personal goal-setting among women living with breast cancer: protocol for a scoping review
Source: Syst Rev. 2018 Aug 28;7:132. doi: 10.1186/s13643-018-0794-6 (PMC6114874; doi:10.1186/s13643-018-0794-6)
Supplement: Supplementary file 2 — Medline search strategy. The search strategy to be used in the MEDLINE database. (DOCX 73 kb) [file 13643_2018_794_MOESM2_ESM.docx]

**MEDLINE SEARCH STRATEGY**

| 1 | exp Breast Neoplasms/ |
| --- | --- |
| 2 | (breast adj3 cancer*).tw. |
| 3 | (breast adj3 carcinoma*).tw. |
| 4 | (breast adj3 neoplasm*).tw. |
| 5 | (breast adj3 tumo?r*).tw. |
| 6 | (mammar* adj3 cancer*).tw. |
| 7 | (mammar* adj3 carcinoma*).tw. |
| 8 | (mammar* adj3 neoplasm*).tw. |
| 9 | 1 or 2 or 3 or 4 or 5 or 6 or 7 or 8 |
| 10 | goals/ or intention/ |
| 11 | (personal adj1 (project* or striving* or goal*)).tw. |
| 12 | (current adj1 concern*).tw. |
| 13 | (life adj1 task*).tw. |
| 14 | (goal* adj5 (set* or establish* or plan* or elicit* or agree* or negotiat* or propos* or develop* or formulat* or elaborat* or identif* or write or written or stat* or specif* or construct* or manag* or direct* or orient* or attain* or achiev* or evalua*)).tw. |
| 15 | (set* adj2 target*).tw. |
| 16 | (goal* adj1 (set* or plan* or attain* or direct* or orient* or cent?r* or assess* or adher* or complian*)).tw. |
| 17 | 10 or 11 or 12 or 13 or 14 or 15 or 16 |
| 18 | 9 and 17 |
